# New records of moth flies (Diptera, Psychodidae) for the Dutch Fauna

**DOI:** 10.3897/BDJ.11.e108636

**Published:** 2023-09-18

**Authors:** Santiago Jaume-Schinkel, Gunnar Mikalsen Kvifte, Iva Njunjić, Menno Schilthuizen

**Affiliations:** 1 Museum Koenig, Bonn, Germany Museum Koenig Bonn Germany; 2 Nord University, Steinkjer, Norway Nord University Steinkjer Norway; 3 Naturalis Biodiversity Center, Leiden, Netherlands Naturalis Biodiversity Center Leiden Netherlands; 4 Taxon Expeditions B.V., Leiden, Netherlands Taxon Expeditions B.V. Leiden Netherlands; 5 Leiden University, Leiden, Netherlands Leiden University Leiden Netherlands

**Keywords:** citizen science, Psychodinae, Taxon Expeditions, dark taxa, COI barcoding

## Abstract

**Background:**

Prior to this study, the moth flies in The Netherlands were represented by 61 species. Our findings derive from a citizen-science expedition in the Vondelpark in Amsterdam, one of the oldest public parks and best known parks in The Netherlands. The combination of citizen science and the exploration of a well-known urban park has allowed us to contribute to the knowledge of moth fly species present in The Netherlands. The findings from this study provide valuable insights into the distribution, taxonomy and genetic resources of *Psychoda* and *Panimerus* species, enhancing our understanding of insect biodiversity and promoting future research in this field.

**New information:**

Our study provides two new geographical records of the moth flies in The Netherlands, namely, *Psychodauniformata* Haseman, 1907 and *Panimerusmaynei* (Tonnoir, 1920) elevating the total number of species to 63. Furthermore, we provide re-descriptions of the females of *Panimerusnotabilis* (Eaton, 1893) and *P.goetghebueri* (Tonnoir, 1919). Additionally, we make available for the first time, the sequence of the 5’-end of the cytochrome c oxidase subunit I (*COI*) gene or *COI* Barcodes for *Panimerusnotabilis*, *P.goetghebueri* and *P.maynei*. These *COI* Barcodes serve as valuable tools for future species identification within the genus.

## Introduction

Moth flies (Diptera, Psychodidae) exhibit a global distribution and have been classified into approximately 3,000 documented species (Pape et al. 2011, Curler et al. 2019). This widespread family showcases remarkable diversity, particularly in tropical regions, while Europe alone has documented more than 500 species (Wagner 2013). Despite the relatively extensive taxonomic knowledge of European Psychodidae, ongoing efforts continue to reveal new species and new geographical records (e.g. Wagner and Kvifte (2015), Omelková and Ježek (2017), Morelli (2018), Kvifte et al. (2020), Jaume-Schinkel et al. (2022)Jaume-Schinkel et al. (in press)). However, there are gaps in the species distributions and knowledge remains limited; regional surveys consistently provide previously undocumented distributional records (e.g. Kvifte (2019), Jaume-Schinkel et al. (2022), Morelli and Biscaccianti (2022)).

The first checklist for the Dutch Psychodidae fauna was published in the early 1930s (Barendrecht 1934) listing 34 species for the country. Later, Wagner and Beuk (2002) published an updated checklist with 48 species. In recent years, several new additions to the Dutch fauna have been made (e.g. Boumans (2009a), Boumans (2009b), Cuppen (2009), Boumans (2011), Omelková and Ježek (2017), Ciliberti (2017), Ciliberti et al. (2017), Beuk (2021), Wagner and Beuk (2021)) bringing the total number of species to 61.

During a citizen-science entomological survey conducted in The Netherlands (Amsterdam), a Malaise trap was set in an ecologically-managed portion of one of the city's oldest parks (Vondelpark) (see van Achterberg et al. (2020), Schilthuizen et al. (2021). Derived from this survey, we recorded for the first time two species in The Netherlands bringing the total number of species to 63. Moreover, we re-describe the females of *Panimerusnotabilis* (Eaton, 1893) and *P.goetghebueri* (Tonnoir, 1919) and we discuss the morphological characters to distinguish the known females of the genus. Additionally, we make available for the first time the *COI* Barcodes (sequence of the 5’-end of the cytochrome *c* oxidase subunit I (*COI*) gene) for *Panimerusnotabilis*, *P.goetghebueri* and *P.maynei*.

## Materials and methods

### Terminology

We follow the general terminology proposed by Cumming and Wood (2017).

### Collection and preparation of specimens

Specimens of moth flies were collected using a Malaise trap during a citizen-science ‘Taxon Expedition’ (for the term, see Schilthuizen et al. (2017) and http://www.taxonexpeditions.com). The expedition was conducted in an ecologically-managed part (the ‘Koeienweide’) in one of the oldest and best-known parks in Amsterdam (Vondelpark, opened in 1865). Specimens were euthanised and preserved in 70% ethanol.

In the material examined section, at the end of each record, the holding institution is given between square brackets ([]). The abbreviations used for collections and their equivalents are given below:

ZFMK: Museum Koenig, Leibniz-Institut zur Analyse des Biodiversitätswandels (previously known as Zoologisches Forschungsmuseum Alexander Koenig), Bonn, Germany.

TXEX: Taxon Expeditions collection, Leiden, Netherlands.

### Genetics

A non-destructive methodology for DNA extraction from complete specimens was performed in the facilities of Museum Koenig following the procedure detailed in Jaume-Schinkel and Mengual (in press). Specimen slide preparation was done following the protocol explained by Jaume-Schinkel and Kvifte (2022), modifying it using the whole specimen for DNA extraction. In the examined material section, the GenBank accession numbers for each specimen are given between brackets ().

Furthermore, for the genus *Panimerus*, we downloaded and used all the available sequences from BOLD (www.boldsystems.org) to include in the *COI* tree and we used Geneious Prime ver. 2022.1.1 (Biomatters, Auckland, New Zealand) to perform a distance-based neighbour-joining (NJ) analysis using the Jukes-Cantor model. On the *COI* tree, the name for each specimen contains the following information: name of the species | BOLD accession number | sample ID | GenBank accession number.

Additionally, *COI* barcodes of *Psychodauniformata* were submitted to the BOLD Identification System (IDS) for animal identification using *COI* sequences and compared with the published sequences by Gibernau and Albre (2022). All sequences can be accessed in BOLD under the Dataset DS-TEPANI (available: https://doi.org/10.5883/DS-TEPANI).

## Taxon treatments

### Panimerus albifacies

(Tonnoir, 1919)

D3434509-AFC8-5159-8C97-52026648CD69

Pericoma albifacies Tonnoir, 1919. Tonnoir (1919): 12. TL. Belgium, Brussels.
Telmatoscopus albifacies Tonnoir: Freeman (1950): 86, Jung (1956): 181, Duckhouse (1962): 419, Vaillant (1972): 69, Withers (1989): 32
Telmatoscopus (Panimerus) albifacies Tonnoir: Tonnoir (1940): 22, Vaillant (1961): 135.
Panimerus albifacies Tonnoir: Krek (1999): 152.

#### Description

##### Figures

Fig. 1 A-C

##### Examined Material

1 ♀. The Netherlands, Amsterdam, Vondelpark 52.3578°N, 4.8671°E. 19.VII.2019-27.VII.2019. Leg. Taxon Expeditions Team, ZFMK-TIS-2638055 (OR139013) [ZFMK] ; 1 ♂ same data as preceding, except 3.VI.2019-12.VI.2019. Leg. van der Meer, Marrit, ZFMK-TIS-2638076 (OR139014) [ZFMK] ; 2 ♂♂ same data as preceding, except 21.VI.2019-25.VI.2019. ZFMK-TIS-2638086 (OR139004), ZFMK-TIS-2638094 (OR139007) [TXEX].

##### Diagnosis

**Figure 1.**
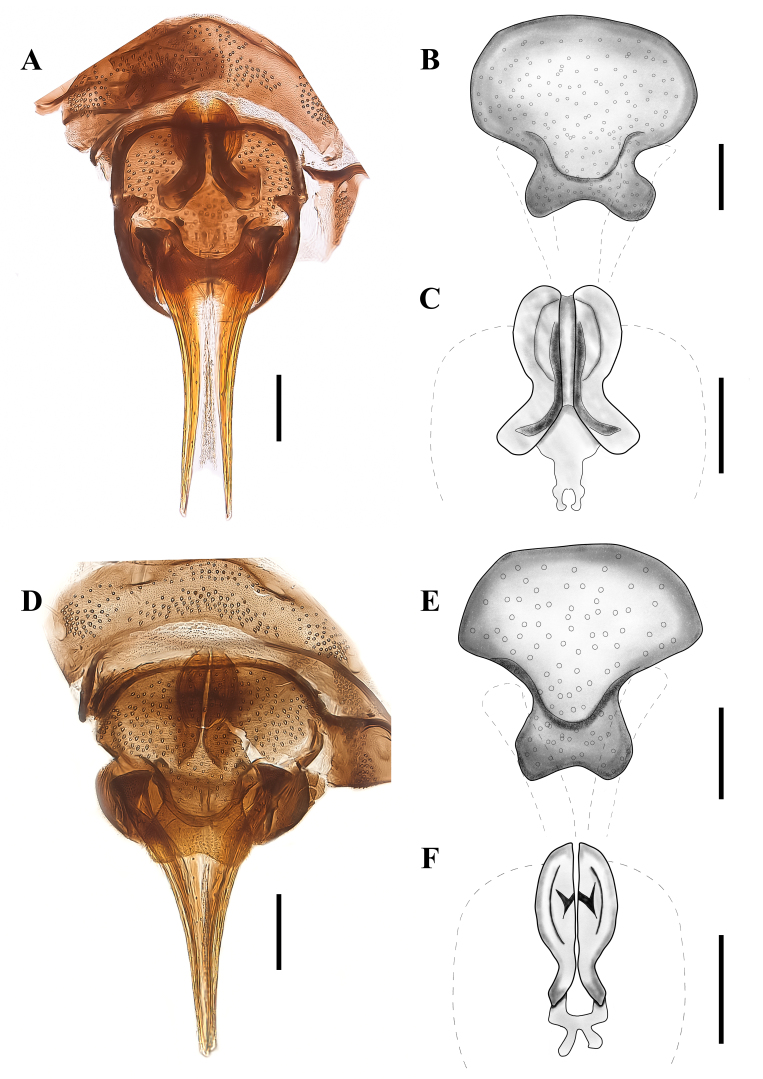
**Figure 1. A-C** *Panimerusalbifacies* female: **A**. Genitalia, **B**. Subgenital plate, **C.** Genital chamber. **D-F** *Panimerusgoetghebueri* female: **D.** Genitalia, **E.** Subgenital plate, **F.** Genital chamber. All scale bars equal 0.10 millimetres.

Females of *P.albifacies* can be easily differentiated from the known females of the genus by the shape of sternite 8 and the shape of the genital chamber Fig. 1 (A-C). Males can be easily differentiated from other males of the genus by the presence of 9 apical tenacula in the surstyli (8, 9 or more than 20 in other species), the hypandrium setose and by the ejaculatory apodeme angular laterally and rounded anteriorly (as in Vaillant (1972): plate IX).

##### Female redescription

Sternite 8 (subgenital plate) is wider than its length, with the anterior margin being 2.5 times wider than the posterior margin, it is covered in small setae with a few scattered larger setae on the dorsal surface, two lateral concavities right before the posterior margin, forming two lobes separated by a concavity in the posterior margin. The cerci are longer than sternite 8. The genital chamber is symmetrical as in Fig. 1A-C.

Based on the male description by Tonnoir (1919) and Jung (1956), the female is similar to the male, except the eye bridge is separated by eight facet diameters; the head is without corniculi; the pedicel is symmetrical; the flagellomeres are smaller than those of the male; apical antennal flagellomeres are absent in examined material.

##### Genetics

four specimens were successfully sequenced: ZFMK-TIS-2638055 (OR139013), ZFMK-TIS-2638076 (OR139014), ZFMK-TIS-2638086 (OR139004) and ZFMK-TIS-2638094 (OR139007). The maximum intraspecific uncorrected pairwise distance for COI sequences was 0.31% or 1 bp.

#### Distribution

Belgium, Bosnia, Czech Republic, Denmark, Estonia, Finland, France, Germany, Hungary, Ireland, Lithuania, Romania, Switzerland, The Netherlands and Turkey (Vaillant 1972, Krek 1972, Salmela and Piirainen 2005, Bernotienė and Rimšaitė 2009, Ježek 2009, Wagner 2013, Wagner et al. 2013, Ježek et al. 2021).

#### Notes

Tonnoir (1919) stated that the female of *P.albifacies* (Tonnoir, 1919) differs from the female of *P.goetghebueri* by the colouration of the setae on the thorax, head and the base of the antennae (with white spots in *P.albifacies* and mainly black in *P.goetghebueri*). However, Freeman (1950) stated that the female of *P.notabilis* is indistinguishable from those of *P.albifacies* and *P.goetghebueri*. Later, Jung (1956) provided a diagnosis and an illustration of the female of *P.albifacies* (Jung 1956: fig. 238). Based on the published female diagnosis, the drawings provided by Jung (1956) and the re-descriptions and figures herein, the females of *P.albifacies* and *P.goetghebueri* can be differentiated by the shape of sternite 8 (anterior margin being 2.5 times wider than the posterior margin in *P.albifacies* and anterior margin 4 times wider than posterior in *P.goetghebueri*); the shape of the genital chamber (*P.albifacies*, Fig. 1 A-C and *P.goetghebueri*, Fig. 1 D–E). However, there is still a large gap when it comes to the description of females of the genus *Panimerus* and further studies could provide better diagnostic characters to easily differentiate the female specimens. Meanwhile, COI barcodes can be useful when it comes to delimiting undescribed females and associating them with previously described and barcoded male specimens.

Vaillant (1972) female diagnosis refers to the female description of Tonnoir (1919).

### Panimerus goetghebueri

(Tonnoir, 1919)

6C2C1B07-416F-58AD-96D5-71EED3941F61

Pericoma goetghebueri Tonnoir, 1919. Tonnoir (1919): 138. TL: Belgium. Gand et Destelberghen
Telmatoscopus goetghebueri Tonnoir: Freeman (1950): 86, Withers (1989): 32.
Panimerus (Panimerus) goetghebueri Tonnoir: Vaillant (1972): 71.
Panimerus goetghebueri Tonnoir: Jezek (1987): 227.

#### Description

##### Figures

Fig. 1 D-F

##### Examined material

1 ♀. The Netherlands, Amsterdam, Vondelpark 52.3578°N, 4.8671°E. 19.VII.2019-27.VII.2019. Leg. Taxon Expeditions Team. ZFMK-TIS-2638058 (OR139011) [ZFMK]; 1 ♂ same data as preceding except for 3.VI.2019-12.VI.2019. Leg. Van der Meer, Marrit. ZFMK-TIS-238056 (OR139010) [TXEX].

#### Diagnosis

Females of *P.goetghebueri* can be easily differentiated from the known females of the genus by the shape of sternite 8 and the shape of the genital chamber (Fig. 1 D and F). Males can be easily differentiated from other males of the genus *Panimerus* by having, at most, eight tenacula on the apex of the surstyli (nine or more in other species) and the ejaculatory apodeme narrowly rod-like (smaller than other species).

##### Female redescription

Sternite 8 (subgenital plate) is wider than its length, with the anterior margin being four times wider than the posterior margin, covered in small setae with a few scattered larger setae on the dorsal surface, two lateral concavities right before the posterior margin, forming two lobes separated by a concavity in the posterior margin. Cerci are about the same length as sternite 8. The genital chamber is symmetrical as in Fig. 1D and F.

Based on the male description of Tonnoir (1919) and Vaillant (1972), the female is similar to the male, except the eye bridge is separated by four facet diameters; the head is without corniculi; the pedicel is symmetrical; the flagellomeres are smaller than those of the male. In the examined material, only the first palpal segment is present; apical antennal flagellomeres are absent as well.

##### Genetics

Two specimens were successfully sequenced: ZFMK-TIS-2638056 (OR139010) and ZFMK-TIS-2638058 (OR139011). The maximum intraspecific uncorrected pairwise distance for COI sequences was 0.45% or 3 bp.

#### Distribution

Algeria, Czech Republic, Hungary, The Netherlands, Tunisia and the UK (Ciliberti et al. 2017).

### Panimerus maynei

(Tonnoir, 1920)

EAAB14B1-AE29-5B1A-AD66-A0F89B3DA75A

Pericoma maynei Tonnoir, 1920. Tonnoir (1920): 186. TL: Belgium, Ohain, Brabant.
Mormia thienemanni Tonnoir: Vaillant (1954): 91. TL: Algeria, Tala Guilef.
Telmatoscopus maynei Tonnoir: Nielsen (1961): 140.
Panimerus (Panimerus) maynei Tonnoir: Vaillant (1972): 72.

#### Description

##### Examined material

1 ♂. The Netherlands, Amsterdam, Vondelpark 52.3578°N, 4.867°E. 13.VI.2019-12.VI.2019. Leg. Van der Meer, Marrit. ZFMK-TIS-2638072 (OR139009) [TXEX]. 1 ♂ same data except for 21.VI.2019-25.VI.2019. ZFMK-TIS-2638090 (OR139001) [ZFMK].

##### Diagnosis

Females of *P.maynei* are unknown. Males can be easily differentiated from other species in *Panimerus* by having more than 30 tenacula on the surstyli (less than 20 in other species), the distribution of the tenacula being scattered in the whole surface of the surstyli (other species in the genus have the tenacula restricted to the apex of the surstyli).

##### Genetics

Two specimens were successfully sequenced: ZFMK-TIS-2638072 (OR139009) and ZFMK-TIS-2638090 (OR139001). Both obtained sequences are identical.

#### Distribution

Belgium, Czech Republic, Denmark, France, Germany, Ireland, The Netherlands (this publication, new record) and the UK. (Vaillant 1972, Withers 1989, Vaillant and Withers 1992).

### Panimerus notabilis

(Eaton, 1893)

476BBC5F-943E-55AD-81D6-94F915FA4216

Pericoma notabilis Eaton, 1893. Eaton (1893): 126. TL. Great Britain.
Telmatoscopus notabilis Eaton: Tonnoir (1919): 12; Freeman (1950): 86, Duckhouse (1962): 418, Withers (1989): 32.
Telmatoscopus (Panimerus) notabilis Eaton: *Tonnoir (1940)*: 28, Jung (1956): 179.
Panimerus (Panimerus) notabilis Eaton: Vaillant (1972): 68.
Panimerus notabilis Eaton: Krek (1972): 184, Wagner (1979): 42, Ježek (1982): 52, Ježek (1984): 165, Jezek (1987): 237. (See Jezek (1987) for a complete taxonomic history).

#### Description

##### Examined material

1 ♂ The Netherlands, Amsterdam, Vondelpark 52.3578°N, 4.8671°E. 03.VI.2019-12.VI.2019. Leg. Van der Meer, Marrit ZFMK-TIS-2638082 (OR139012) [TXEX]. 1 ♂ same data as preceding, except for 12.VII.2019-19.VII.2019, ZFMK-TIS-2638117 (OR139000) [ZFMK].

##### Diagnosis

Females of *P.notabilis* are unknown. Males can be easily differentiated from all the males of the genus *Panimerus* by having nine apical tenacula in the surstyli (8, 9 or more than 20 in other species), the ejaculatory apodeme with rounded lateral lobes and is concave anteriorly (Vaillant 1972: plate IX) and by the shape of the aedeagal complex (as in Withers (1989): fig. 96).

##### Genetics

Two specimens were successfully sequenced: ZFMK-TIS-2638117 (OR139000) and ZFMK-TIS-2638082 (OR139012), these barcodes corresponding to the first barcodes of the species. The maximum intraspecific uncorrected pairwise distance for COI sequences was 0%.

#### Distribution

Belgium, Croatia, Finland, France, Germany, Hungary, Iran, Ireland, Italy, Poland, Romania, The Netherlands and Turkey (Ježek and Omelková 2012, Ježek et al. 2018, Wagner 2013, Wagner et al. 2013, Kvifte et al. 2013).

### Psychoda uniformata

Haseman, 1907

61AA11D3-D1C4-5CBF-9184-06ACA5181162

Psychoda uniformata Haseman, 1907. Haseman (1907): 319. TL: USA. Missouri: Columbia.
Psychoda moravica Vaillant, 1966. Vaillant (1966): 225. TL: Czech Republic, Praděd (see Ježek (1990)).
Psychoda uniformata Haseman: Ježek (1990): 67.

#### Description

##### Examined material

1 ♀. The Netherlands, Amsterdam, Vondelpark 52.3578°N, 4.8671°E. 27.V.2019-5.VI.2019. Leg. Taxon Expeditions Team. ZFMK-TIS-2638051 (OR139003) [ZFMK]; 1 ♀ same data, except for 3.VI.2019-12.VI.2019. Leg. Van der Meer, Marrit. ZFMK-TIS-2638081 (OR139015) [TXEX].

#### Diagnosis

Females of *P.uniformata* can be differentiated from other *Psychoda* species by the shape of sternite 8 (subgenital plate) (as in Ježek (1990) fig. 152) and the shape of the genital chamber (as in Ježek (1990) fig. 145).

Males can be distinguished from other *Psychoda* species on the following combination of characters: the antennae with 13 flagellomeres; the gonostyli apically pointed, the distiphallus is broadly triangular, narrowing towards apex; a single paramere is present, reaching more than four-fifths length of the distiphallus. *Psychodauniformata* is similar to *Psychodacultella* Salmela, Kvifte & More, 2012 and *Psychodaobscuripennis* Jezek & van Harten, 2005, but they can be differentiated by the following characters: the antennae with 13 flagellomeres (14 in *P.cultella*, 13 in *P.obscuripennis*); the gonostyli are apically pointed (apically pointed in *P.cultella* and club-shaped in *P.obscuripennis*); the distiphallus broadly triangular (distiphallus parallel-sided in both *P.cultella* and *P.obscuripennis*); the paramere reaching more than four-fifths the length of the distiphallus (paramere subequal in length to the distiphallus in *P.cultella*, paramere reaching roughly two-thirds the length of the distiphallus in *P.obscuripennis*) (Ježek 1990, Ježek and van Harten 2005, Salmela et al. 2012).

##### Genetics

Two specimens were successfully sequenced: ZFMK-TIS-2638051 (OR139003) and ZFMK-TIS-2638081 (OR139015). The maximum intraspecific uncorrected pairwise distance for COI sequences was 2.12% or 14 bp.

#### Distribution

Armenia, Austria, Azerbaijan, Czech Republic, Greece, Iran, Israel, Italy, Slovakia, Slovenia, Mongolia, Morocco, The Netherlands (this publication, new record), Poland, Turkey, USA (Ježek et al. 2021, Gibernau and Albre 2022).

## Discussion

Citizen-science projects can provide a more accurate picture of the real distribution of species. Previous studies by Maistrello et al. (2016), Alaniz et al. (2018), Dörler et al. (2018), Barahona-Segovia and Barceló (2021), Jaume-Schinkel and Mengual (2022), Kvifte (2023), Jaume-Schinkel et al. (in press) have highlighted the importance of citizen science in capturing species distribution data. In our study, we found two new records for The Netherlands through a citizen-science project, adding valuable information to the existing knowledge base.

These findings demonstrate the power of citizen-science initiatives in uncovering previously unknown distribution patterns and expanding our understanding of species ranges (Barahona-Segovia et al. 2022, Jaume-Schinkel and Mengual 2022, Jaume-Schinkel et al. in press). The integration of citizen-science initiatives has proven to be an invaluable asset in advancing our understanding of species distribution patterns (Barahona-Segovia and Barceló 2021). By engaging and involving the general public in scientific research, citizen-science projects provide a vast network of enthusiastic and motivated individuals who contribute to data collection on a scale that would be otherwise impossible for traditional research teams (Gardiner et al. 2012, Johnson et al. 2020, Mengual and de Soto Molinari 2020, Feldman et al. 2021, Howard et al. 2022).

In addition to their scientific contributions, citizen-science projects foster public engagement and awareness of biodiversity. By involving citizens in scientific research, these projects not only empower individuals, but also enhance their understanding of ecological processes and the importance of conservation efforts. Participants in citizen-science initiatives become ambassadors for the natural world, advocating for the preservation of species and their habitats.

**Figure 2.**
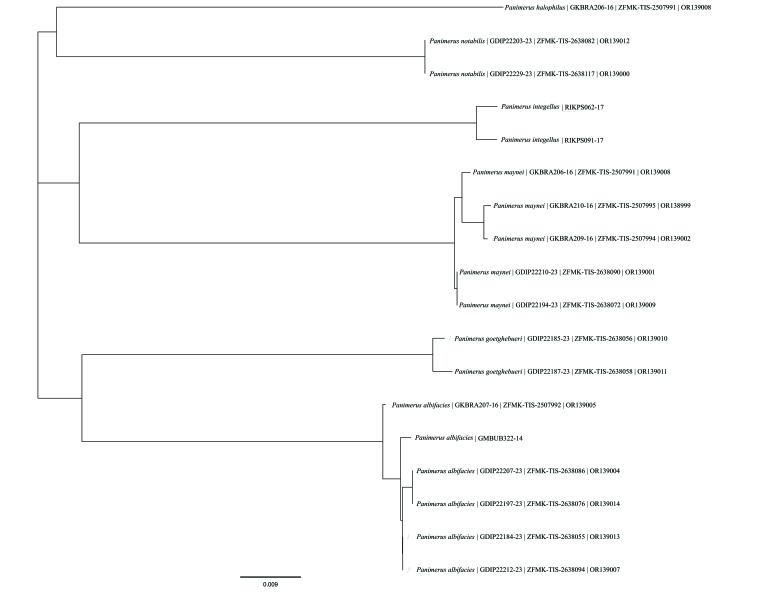
Neighbour-joining (NJ) tree using the Jukes-Cantor model, based on the *COI* sequences of the examined material and publicly-available sequences. NJ tree constructed using Geneious Prime ver. 2022.1.1. The name for each specimen has the following information: name of the species | BOLD accession number | sample ID | GenBank accession number.

Additionally, our study demonstrates the effectiveness of *COI* barcodes as a valuable tool for species identification within the genus *Panimerus* (Fig. 2). This approach greatly aids taxonomists in associating male and female specimens when there is a lack of distinct morphological features to establish a connection between both sexes. Moreover, DNA barcodes simplify the process of matching specimens from different sexes, especially in cases where only one sex is known. This streamlined matching process contributes to the identification and description of new morphological characters, which are often overlooked when working solely with one sex (such as relying heavily on male genital characters in taxonomy).

Moreover, further investigation into the applications of DNA barcoding, such as the use of other genetic markers or the integration of genomic techniques, could provide even more robust and comprehensive insights into species delimitation and distributions. It would be worthwhile to explore the potential of combining DNA barcoding with other data sources, such as remote sensing or environmental DNA, to gain a more holistic understanding of species distributions and their drivers.

## Supplementary Material

XML Treatment for Panimerus albifacies

XML Treatment for Panimerus goetghebueri

XML Treatment for Panimerus maynei

XML Treatment for Panimerus notabilis

XML Treatment for Psychoda uniformata
